# Pushing the limit: Examining factors that affect anoxia tolerance in a single genotype of adult *D. melanogaster*

**DOI:** 10.1038/srep09204

**Published:** 2015-03-17

**Authors:** Raquel Benasayag-Meszaros, Monica G. Risley, Priscilla Hernandez, Margo Fendrich, Ken Dawson-Scully

**Affiliations:** 1 Florida Atlantic University, Department of Biological Sciences, 5353 Parkside Drive, Jupiter, FL. 33458, USA

## Abstract

*Drosophila melanogaster* is a promiscuous species that inhabits a large range of harsh environments including flooded habitats and varying temperature changes. To survive these environments, fruit flies have adapted mechanisms of tolerance that allow them to thrive. During exposure to anoxic stress, fruit flies and other poikilotherms enter into a reversible, protective coma. This coma can be manipulated based on controlled environmental conditions inside the laboratory. Here we utilize a common laboratory raised strain of *D. melanogaster* to characterize adaptation abilities to better understand coma recovery and survival limitations. Our goal is to mimic the fly's natural environments (wet anoxia) and relate findings to a typical gas induced environment (dry anoxia) that is commonly used in a laboratory. Despite the abundance of research regarding acute and chronic anoxic exposure and cold stress, the literature is lacking evidence linking anoxic stress with variable environmental conditions such as animal age and stress duration. We present novel ways to assess coma recovery and survival using readily available laboratory tools. Our findings suggest that younger age, exposure to colder temperatures and wet environments increase resistance to anoxic stress.

D*rosophila melanogaster*, the common fruit fly, has adapted to inhabit almost every continent in the world^1^,^2^. Through microevolution, fruit flies have evolved superior mechanisms to help cope with constantly changing temperatures and fluctuating rainfall as a result of global climate change within their narrowly adapted niches^3^. It's critical to study how organisms are going to contend with both long term climate fluctuations and short term conditions such as flooding and freezing^4^. As poikilotherms, *D. melanogaster*'s internal temperature is directly mirrored by the ambient environment making the species an ideal model to study survival mechanisms during extreme weather. Although the animals used in this study are not collected directly from the field, they are genetically identical and therefore ideal for examining these parameters in the laboratory. Utilizing a universally available, inbred strain previously established in the laboratory allows for significantly reduced behavioral variability and complements previous experiments on stress tolerance using laboratory adapted insects^5^.

While the promiscuity of this species makes it a perfect model to study adaptation abilities both in a natural environment and in a laboratory setting, current literature is missing the direct link between the common method of inducing anoxia in a laboratory, by gaseous anoxia, to that of natural anoxic conditions, such as flooding^6^. Previous work established the paradigm of anoxia tolerance, or the ability to withstand zero oxygen, during water submersion-induced anoxia in three spider species^7^. Salt-marsh and forest inhabiting wolf-spider species were submerged in water to simulate habitat flooding. The salt-marsh spider species demonstrated an ability to enter into a non-reactive, coma-like state^7^. Similarly, when a fruit fly is exposed to a comparable anoxic environment it enters into a reversible hypometabolic coma and recovers without pathology when normal oxygen levels are restored^8^,^9^. A comparable, behavioral phenomenon has also been observed when insects are exposed to cold temperature^10^. *D. melanogaster* will enter into a characteristic cold-induced chill-coma and recover movement when introduced to permissible temperature^11^,^12^. An additional factor important to recognize is the potential of desiccation stress during droughts and climate fragmentation in nature and gaseous anoxia laboratory experiments^13^,^14^. While flies have been exposed to cold and gaseous anoxic environments in the past, the effects of water, age, and exposure duration in universally available wild type laboratory strains of *D. melanogaster* have yet to be cohesively interrelated.

Additionally, literature is missing data connecting the detrimental effects of increased reactive oxygen species (ROS) production and protein oxidation during aging with stress tolerance^15^,^16^,^17^. When insects are re-exposed to normoxic environments following bouts of anoxia, oxygen is reintroduced to cells and ROS begin to oxidize biomolecules important for survival. Over time, these damaged molecules lead to cell and eventually animal death. Similarly, as an insect ages ROS begin to slowly build up, leading to an increased amount of cell damage when compared to younger insects^17^,^18^. It is important to develop a thorough understanding of how the effects of age, temperature, and type of anoxic environment can influence anoxia tolerance in terms of both recovery and survival while focusing particular attention on comparing laboratory induced gaseous anoxia to wet anoxia in *D. melanogaster*'s natural habitat.

Taking this into account, we present a novel method to assess the limitations of recovery and survival of *D. melanogaster* with the consideration of four important factors: 1) anoxic environment (submersion vs. gaseous), 2) anoxic exposure duration (0 to 72 h), 3) ambient temperature (23°C vs. 3°C), and 4) age (young vs. old). By developing a novel assay to simulate flooded habitats and temperature fluxes we will cohesively define the limits of anoxic stress, temperature, environment, and age in *D. melanogaster* and provide a necessary missing link in ecology literature.

## Results

### Age and anoxia tolerance

**Figure 1:**
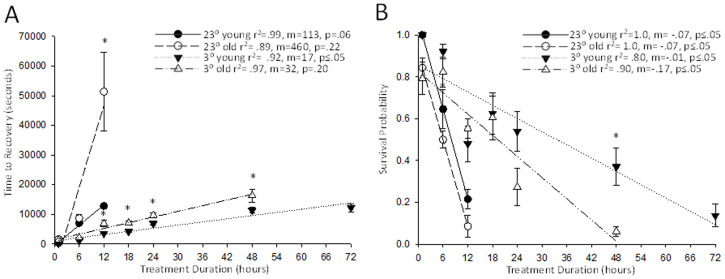
Young (one to nine days) and old (35–39 days) adult *D. melanogaster* were submerged for a designated duration at 23°C and 3°C and were then dried and allowed to recover. A) Average time to recovery was recorded after a designated duration of submersion anoxia. Both young and old *D. melanogaster* submerged at 3°C took significantly less time to recover than flies submerged at 23°C. Flies submerged at 23°C did not survive after a submersion time of 12 h whereas flies submerged at 3°C survived up to 72 h of submersion. B) Survival was assessed at 24 h after flies were removed from the submersion chambers. Flies submerged after 12 h at 23°C did not survive. All time points are shown as mean ± SEM and significant differences are represented with asterisks denoting a significant difference to the time points below the asterisk where P < 0.05.

**Figure 2:**
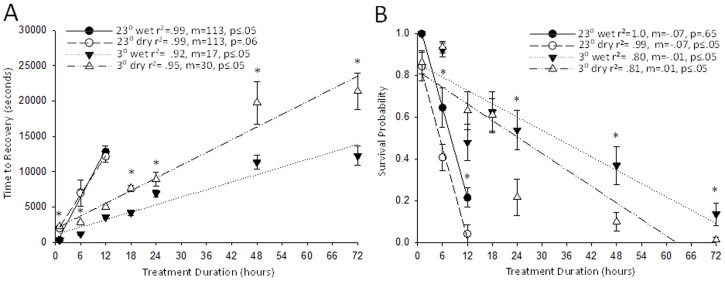
Adult *D. melanogaster* were exposed to wet and dry anoxia for a designated duration and allowed to recover. A) Average time to recovery was recorded after a designated duration of wet and dry anoxia at 23°C and 3°C. Adult *D. melanogaster* exposed to wet anoxia at 3°C took significantly shorter time to recover than flies exposed to either temperature of dry anoxia. Flies exposed at 23°C did not survive after 12 h of anoxia whereas flies exposed at 3°C survived up to 72 h of exposure. B) Survival was assessed at 24 h after flies were removed from anoxia. Flies exposed after 12 h of wet and dry anoxia duration at 23°C did not survive. All time points are shown as mean ± SEM and significant differences are represented with asterisks denoting a significant difference to the time points below the asterisk where P < 0.05.

We assessed the role aging plays in regard to stress tolerance in a wet environment at room temperature (23°C) and cold temperature (3°C) and revealed that older animals take longer to recover, especially at 23°C, and have a lower probability of survival. There was a significant difference between the recovery times of old and young flies submerged at 23°C for 12 h (N > 9; three-way ANOVA, *F*_(2,585)_ = 41.527; P < 0.001;Holm-Sidak, P < 0.001; Fig. 1A). At 3°C, older flies took longer to recover at every time point after 12 h of submersion, (Holm-Sidak, P < 0.015) indicating age as an important physiological factor for anoxic stress tolerance. All regression lines corresponding to both figures 1 and 2 had r^2^ values greater than 0.79, suggesting data fit the model closely. Survival data disclosed an interesting trend at room temperature, old and young flies only survived up to 12 h, while at the cold temperature, young flies survived up to 72 h of stress and old flies survived 48 h of stress (three-way ANOVA,N > 9, *F*_(2,53)_ = 0.673, P > 0.003; Fig. 1B). During submersion insults at cold temperatures for 1–24 h, there was no significant difference between survival rates among age groups. While room temperature conditions displayed overall significant differences through analysis with three-way ANOVA (N > 9, *F*_(2,53)_ = 0.673, P < 0.011).

### Linking environment, age, stress duration, and temperature

The previous submersion experiments were intended to imitate flooded habitats in nature, but it is important to compare these to typical gas induced anoxia environments created in a laboratory to account for potential additional stressors. Examining the recovery time of flies subjected to 23°C gaseous and submersion protocols indicate a significant difference only after 1 h of exposure, and not after 6 h and 12 h of exposure (N > 7; three-way ANOVA, *F*_(2,674)_ = 2.11, P < 0.001; Holm-Sidak, P < 0.007; Fig. 2A). At cold temperature, all the stress periods revealed a significant difference in the recovery times between the wet and dry assays except for the 12 h duration, with the gaseous anoxia showing consistently longer recovery times (Holm-Sidak, P < 0.039). These results coincide with the trends observed throughout the experiment; cold temperature and submersion anoxia increase stress tolerance in adult *D. melanogaster.* The survival data collected reveals interesting trends. While flies were tested at room temperature past 72 h, animals did not recover after 12 h. At room temperature, all time points were significantly different between the submersion and anoxic chamber experiments but at cold temperatures, there is no significant difference until 24 h of exposure (three-way ANOVA, *F*_(2,65)_ = 2.118, P < 0.003; Fig. 2B).

## Discussion

In its natural environment, *Drosophila* are occasionally exposed to variable ecological conditions that test the limits of their survival^1^,^3^. Past work investigating anoxia in laboratories has focused on acute and chronic effects of anoxic exposure but evidence linking this environment to typical anoxia in nature, while tying in age and temperature influences is lacking. This investigation characterizes anoxia tolerance both in a wet, submerged environment and a dry, gaseous environment and relate these to other parameters that are prevalent for stress tolerance, specifically: age, temperature, and stress duration.

Although flies demonstrate an impressive ability to survive acute bouts of anoxia, there are limitations to survival when anoxic exposure is extended. A possible explanation of these findings relates to the production and depletion of cellular energy during an anoxic coma. During anoxic events, the metabolic rate decreases significantly, allowing the fly to preserve cellular ATP while also significantly decreasing total ATP production^5^,^9^,^19^,^25^. When the fly begins to recover upon reoxygenation, there is less ATP available to restore metabolic deficits, due to ATP depletion during anoxia, and subsequent survival is compromised^19^,^20^,^21^. Additionally, ATP depletion leads to failure of the Na^+^/K^+^ ATPase, leading to dysregulation of ionic homeostasis, protein unfolding and subsequently protein aggregation^22^. Our findings reveal that flies exposed to longer periods of stress take more time to recover from the insult. Previous work assessing anoxic exposure and recovery time reflects similar trends in that there is a strong inverse correlation between increasing stress duration and decreasing survival probability, possibly due to deficits in ATP production and the inability for the animal to compensate for ATP consumption^5^,^6^. It is also a possibility that as the flies are metabolizing O_2_ and producing CO_2_, hypercapnia is aiding to the anoxia coma^23^,^24^. However, we believe this has minimal affects as *Drososphila* spiracles quickly release CO_2_ in hypercapnic environments.

It appears that lowering the temperature inherently protects *D. melanogaster* from anoxic stress. Flies that are subjected to cold anoxic stress take less time to recover from a coma and have a greater survival rate when compared to the flies subjected to room temperature anoxia for both submersion and gaseous environments. This can be explained through Rodriguez and Robertson's (2012) results suggesting that during repetitive anoxia, decreased temperature has a protective effect on regulating neuronal K^+^ homeostasis^25^. Lowering the temperature during repetitive, acute anoxic events leads to a lesser increase in extracellular K^+^, when compared to hyperthermia. Cells are therefore able to return to normal ion homeostatic levels quicker with less cellular damage and an overall increase in their survival probability^25^. Work in the fall field cricket (*Gryllus pennsylvanicus)* concerning chill-coma recovery (CCR) also suggests ion homeostasis in gut epithelial cells plays a role in coma onset and recovery^10^. It is important to note that previous investigations into the effect of anoxia treatment duration (7.5–60 minutes) and temperature (20°C–30°C) on recovery concluded that temperature did not influence recovery time^23^. However, there were critical differences in the methodology, most importantly, the current investigation extends treatment time to 72 h, and decreases temperature to 3°C.

Additionally, our data suggests that regardless of temperature, flies exposed to submersion anoxia recovered quicker and had a higher probability of survival. There are several possible explanations for this affect, 1) water the lessened potential for desiccation stress and 2) the possibility of oxygen in the water. Extensive research concerning desiccation, or dryness, in *Drosophila* as an additional stressor in the ambient environment suggests that *Drosophila*, along with many other temperate weather insects, are highly susceptible to desiccation stress^13^,^26^. Exposure to environmental desiccation potentially generates an additional stressor to which submerged flies are not subjected. Previous investigations have determined that as *Drosophila* are exposed to gaseous anoxic conditions, ATP depletion causes mechanical failure of the spiracle muscles^24^,^27^. This leads to water loss through the spiracle and additional desiccation stress^28^. The paradigm of desiccation stress during recovery time is also reflected when assessing survival probability between dry anoxia and submerged flies. Lastly, in order to mimic a natural ecosystem, oxygen was not removed from water in the submersion container. Therefore, there remains the possibility that oxygen in the water contributed to the more successful recovery times and survival proportions.

According to the oxidative stress theory of aging, as animals progress in age, ROS increase and oxidative stress becomes a factor governing lifespan. During increased stress including anoxia, there is an additional accumulation of ROS^17^,^29^. Oxygen deprivation is accompanied by ROS formation which damages lipids, proteins, and DNA, further promoting cell damage^30^. Previous research suggests that the sum of ROS due to the natural process of aging, in addition to ROS produced under anoxic stress, leads to increased cellular damage, resulting in a longer recovery time and a decreased chance of survival^30^,^31^. Our investigation of the effect of age on anoxia tolerance suggests that older age increases recovery time and decreases survival probability after anoxic stress at every duration greater than 6 h in both cold and room temperatures. This indicates that age plays a major role in stress tolerance.

In summary, we determined that anoxia tolerance is reduced with increased stress exposure duration, increased temperature, and increased age and tolerance is more favorable in wet conditions. At the same time, survival limitations were found to be correlated with these variables. Now that the environmental conditions have been characterized in terms of anoxic stress, they can serve as parameters for future investigation of anoxia tolerance mechanisms. These results filled in specific gaps regarding anoxic tolerance studies in *D. melanogaster* and should now be investigated utilizing the extensive library of genetic tools available for *Drosophila* research to better understand the mechanisms behind anoxia tolerance.

## Methods

### Fly maintenance

*D. melanogaster* w^1118^ stocks were reared at 25°C with 40% humidity on a 12 h:12 h light dark cycle. Flies were raised on 50 mL of standard medium (recipe from Bloomington Stock Center at Indiana University) in plastic culture bottles with approximately 100 adult flies per bottle. All flies used in the experiments were one to nine day old males (designated as young flies) or 35–39 day old males (designated as old flies).

### Submersion assay (wet anoxia)

To simulate the wet environment a container was filled with water and a novel submersion chamber was fabricated. The submersion container was constructed by first cutting the bottom out of a plastic cylinder (approximately 8 cm tall × 6 cm diameter) and replacing the bottom with stiff metallic mesh (1 mm × 1 mm spacing). The top of the submersion container consisted of the same stiff metallic mesh glued to a plastic lid with the center removed. The plastic lid securely fit the dimensions of the cylinder while the center was cut out leaving about a 5 mm overhang in order to have a surface to glue on the metallic mesh. The mesh at the top and bottom of the cylinder allowed for free exchange of water while containing the flies.

After construction of the submersion chamber, young male and old male flies were carefully transferred to separate submersion containers. The containers were placed into the water chamber and tapped against the bottom of the water chamber to eliminate air bubbles trapped in the metallic mesh (Supporting Information (SI), Video S1). As the chambers were submerged, oxygen bubbles breifly encompassed the cuticle but dissipated within 1–2 minutes. Time zero began when the bubble dissipated and fly movement ceased.

To simulate temperature fluxes, the flies were submerged in containers that were either at room temperature (23°C) or cold temperature (3°C). The room temperature experiments were carried out on an undisturbed laboratory bench for the designated amount of time. The water chambers for the cold temperature experiments were monitored with a thermometer and regulated using ice and a refrigerator to maintain 3°C. It was important to ensure flies submerged at 3°C entered into an anoxic coma rather than a cold induced-coma (where oxygen may still remain within the animal); therefore these flies were subjected to argon gas for ten minutes prior to being placed in the submersion container. Submersion chamber immersion in 23°C, removal, re-immersion in 3°C, and removal again seemed to be detrimental to the integrity of the fly wings. Therefore, we elected to use gas to induce the coma and place flies directly into the 3°C submersion container. This process did not alter the time 23°C vs 3°C submerged flies entered into the coma because time zero began at coma onset.

After a specific time interval, the flies were carefully removed using a soft paint brush and placed on a piece of metallic mesh. A Kim wipe (VWR International, Radnor, PA, USA) was placed on the underside of the mesh to wick away the water from the fly without physically touching the fly. Flies were then placed in a plastic food vial and vials were plugged with a foam stopper. The vial was placed on its side to prevent the flies from getting stuck in the food. A digital video recorder was used to record the flies for 24 h post-stress. Time to recovery was defined as the ability to stand up. The recovery time of each fly was recorded; however, ‘N' was the average recovery time of all recovered flies in an individual vial. After 24 h, the number of flies that did not recover was recorded for survival data. (Supporting Information (SI), Video S2).

### Gaseous assay (dry anoxia)

The previous submersion experiments were developed to mimic a natural flooded habitat, but it is important to compare these to a controlled dry anoxic environment (the primary method used in research)^9^,^32^. Male flies one to nine days post eclosion were placed in plastic vials with food. To induce an anoxic coma, the flies were transferred to the submersion containers and placed inside an anoxia chamber containing 90% N_2_, 5% H_2_, and 5% CO_2_ (Air Gas, Miami, FL, USA) along with the materials needed for the next several steps. Inside the anoxia chamber, exposure to the gas mixture caused quick coma onset. This was recorded as time zero. The flies were then transferred into a petri dish with a lid to confine them for the duration of the experiment. A generous portion of wax was placed around the edges of the petri container to ensure complete oxygen isolation. The petri dishes were placed in a vacuum-sealed plastic bag where the remaining oxygen was withdrawn using a food vacuum sealer to prevent oxygen contamination. The petri dishes housing the flies were removed from the anoxia chamber and stored in either a refrigerator to mimic a cold environment (3°C) or on a laboratory shelf at room temperature (23°C). After the designated amount of time, the flies were transferred to a plastic vial with food to record their recovery with a digital video recorder in the same manner as the submerged flies. Because the wet and dry experiments were performed synchronously, the young fly data were combined in the analyses.

### Data Acquisition

Immediately after the flies were transferred into the plastic vials with food, the vials were positioned in front of a digital video camera. The video camera recorded for 24 h. This video was analyzed by recording the exact time when each fly stood upright and began to walk, the average of all flies were recorded as a single N. Survival data was obtained by counting the number of living flies 24 h post stress. The percent of live flies was recorded.

### Statistical analysis

Data was analyzed using three-way analysis of variance (ANOVA) followed by a Holm-Sidak method (Holm-Sidak) pairwise multiple comparisons test using SigmaPlot 11.0 (San Jose, California). Holm-Sidak multiple comparisons test described effects between each variable (ie: only age) while three-way ANOVA describes interactions between all effects. Data were normally distributed. The computer tested our data and it met the assumptions of independence of observations, homogeneous variances, and population normality therefore F value was appropriate to report. In the reported statistics, the F value subscripts represent degrees of freedom and total sample size, respectively. All figures have time points which represent mean ± SEM and regression lines to assess each parameter as an overall function of treatment duration. Figure legends contain all appropriate r^2^ values where values closer to 1.0 represent data best fit by the model. Figures also contain slope (m) and appropriate p values. “N” is defined as one experiment including at least five animals. For example, N > 7 means an average of at least 35 flies was recorded. Significant differences are represented with asterisks denoting a significant difference to the two time points below the asterisk with all P ≤ 0.05.

## Author Contributions

R.B.M. and K.D.S. designed experiments. R.B.M., P.H. and M.F. collected data. R.B.M., M.G.R., P.H., M.F. and K.D.S. analyzed data. R.B.M., M.G.R. and K.D.S. wrote the paper.

## Supplementary Material

Supplementary Information Supporting Information (SI), Video 1

Supplementary Information Supporting Information (SI), Video 2
